# A comparative analysis of particulate bovine bone substitutes for oral regeneration: a narrative review

**DOI:** 10.1186/s40729-024-00544-z

**Published:** 2024-05-27

**Authors:** Andreas Pabst, Philipp Becker, Werner Götz, Diana Heimes, Daniel G.E. Thiem, Sebastian Blatt, Peer W. Kämmerer

**Affiliations:** 1 Department of Oral and Maxillofacial Surgery, German Armed Forces Central Hospital, Rübenacherstraße 170, 56072 Koblenz, Germany; 2 Department of Oral and Maxillofacial Surgery – Plastic Operations, University Medical Center Mainz, Augustusplatz 2, 55131 Mainz, Germany; 3 Department of Orthodontics, University Hospital Bonn, Welschnonnenstr. 17, 53111 Bonn, Germany

**Keywords:** Particulate bovine bone substitute, Oral regeneration, Narrative review, Ridge preservation, Sinus floor elevation, Peri-implant defects

## Abstract

**Purpose:**

Particulate bovine bone substitutes (BS) are commonly used in oral regeneration. However, more literature is needed focusing on comparative analyses among various particulate bovine BS. This study evaluates pre-clinical and clinical data of different particulate bovine BS in oral regeneration.

**Methods:**

A narrative review was conducted by screening the PubMed database Included in the review were pre-clinical and clinical studies until 2024 comparing a minimum of two distinct particulate bovine BS. In addition to examining general data concerning manufacturing and treatment processes, biological safety, physical and chemical characteristics, and graft resorption, particular emphasis was placed on assessing pre-clinical and clinical data related to ridge preservation, sinus floor elevation, peri-implant defects, and various forms of alveolar ridge augmentation utilizing particulate bovine BS.

**Results:**

Various treatment temperatures ranging from 300 to 1,250 °C and the employment of chemical cleaning steps were identified for the manufacturing process of particulate bovine BS deemed to possess biosecurity. A notable heterogeneity was observed in the physical and chemical characteristics of particulate bovine BS, with minimal or negligible graft resorption. Variations were evident in particle and pore sizes and the porosity of particulate bovine BS. Pre-clinical assessments noted a marginal inclination towards favorable outcomes for particulate bovine BS subjected to higher treatment temperatures. However, clinical data are insufficient. No distinctions were observed regarding ridge preservation, while slight advantages were noted for high-temperature treated particulate bovine BS in sinus floor elevation.

**Conclusions:**

Subtle variances in both pre-clinical and clinical outcomes were observed in across various particulate bovine BS. Due to inadequate data, numerous considerations related to diverse particulate bovine BS, including peri-implant defects, must be more conclusive. Additional clinical studies are imperative to address these knowledge gaps effectively.

**Graphical abstract:**

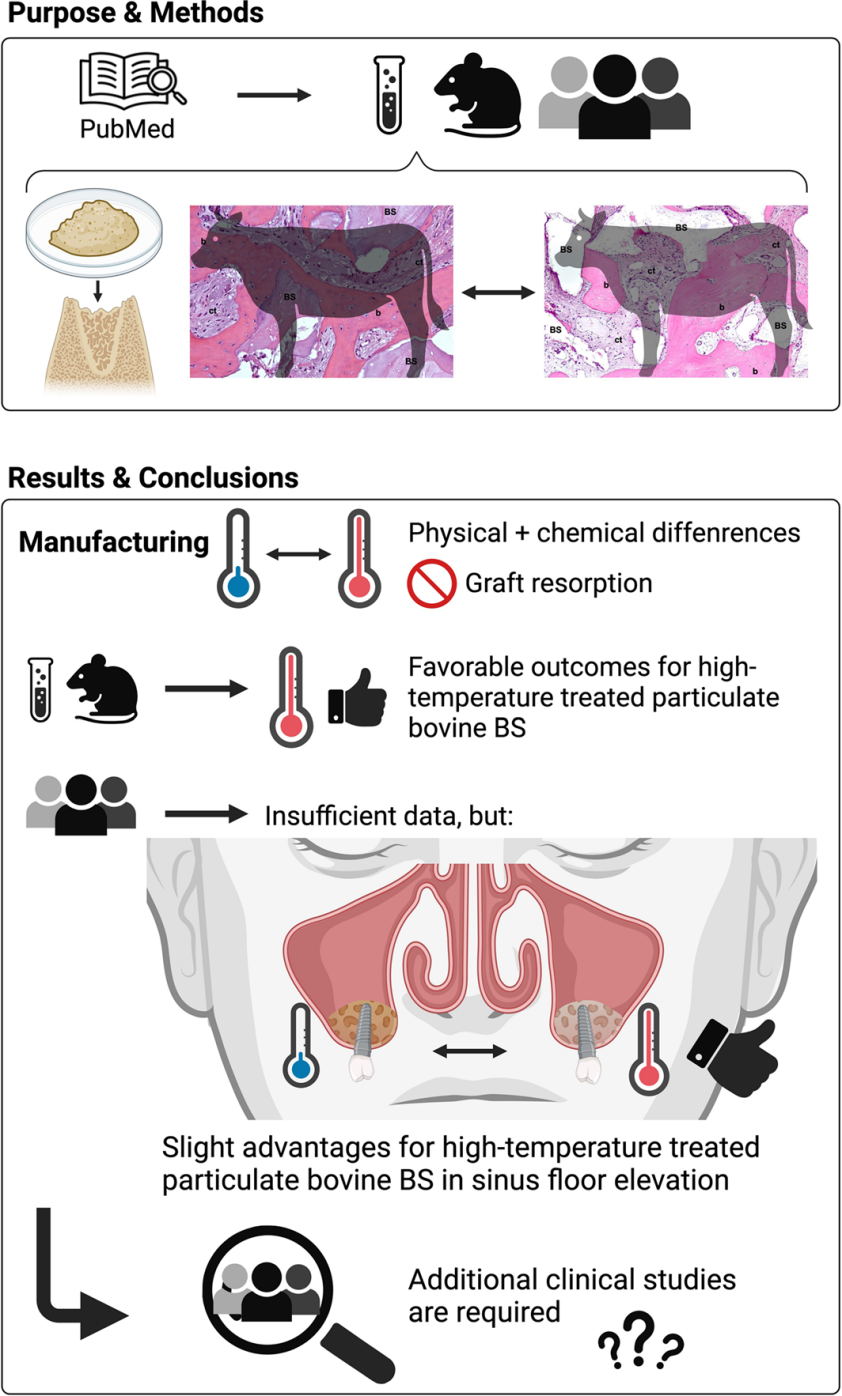

## Background

Dental implantology has grown significantly, emerging as a hallmark of modern dentistry and prosthodontics [1]. A pivotal challenge in this field is ensuring an adequate supply and quality of bone within the alveolar ridge, a critical factor for successful implant placement [2]. A minimum alveolar width of 4 mm and height of 7 mm are deemed necessary for effective implant placement [3]. Unfortunately, the alveolar ridge undergoes continuous three-dimensional atrophy following tooth loss [4], with atrophy rates ranging from 25% within one year to 40–60% within five years of the initial bone volume [5–8]. Consequently, ridge preservation techniques have been established to mitigate this process within specific parameters [9–11].

Moreover, conditions such as craniofacial malformations, periodontal disease, trauma, or post-tumor therapy may further compromise the alveolar bone [12]. Subsequently, short and diameter-reduced implants have been introduced for placement in situations with limited bone availability [13, 14]. Despite these advancements, regeneration of the alveolar crest is often necessary to address alveolar ridge atrophy. Various surgical techniques have been developed, including sinus floor elevation and guided bone regeneration (GBR) [15]. The ideal grafting material should possess osteogenic, osteoinductive, and osteoconductive properties, along with rapid and adequate vascularization and bone remodeling capabilities and high-volume stability to serve as a placeholder until the defect is remodeled by newly formed bone [8, 16]. These prerequisites have led to autogenous bone being recognized as the “gold standard” for alveolar ridge augmentation, owing to its optimal physiological compatibility, expedited healing, and effective integration [17].

Conversely, the drawbacks associated with autogenous bone grafts, including donor site morbidity and potential nerve injuries, are frequently documented [18–21]. As an alternative to autogenous grafts, bone substitutes (BS) of various origins have been developed, including allogeneic (from the same species, human), xenogeneic (from different species, such as bovine, porcine, or equine), and alloplastic (synthetic) materials [11, 22]. Those derived from bovine sources are particularly prevalent among xenogeneic grafts, demonstrating numerous satisfactory long-term clinical outcomes [23, 24]. These bovine-derived BS have exhibited significant biocompatibility and advantageous regenerative potential [25], which could be further improved by biofunctionalization the bovine BS using, for example, platelet-rich fibrin or hyaluronic acid [26–30]. Hence, bovine BS are extensively utilized in regenerative dentistry and oral regeneration, particularly in high- or low-temperature treated forms, such as spongious grafts [31]. Evidence suggests that bovine BS can yield comparable outcomes to autogenous grafts, especially in sinus floor elevation and GBR [16, 32]. This equivalence has been corroborated even regarding implant survival rates following sinus elevation and ridge augmentation using BS [33]. In addition, xenogeneic BS used to preserve the alveolar ridge, especially in conjunction with a barrier membrane, can significantly reduce additional bone grafting procedures. On the other hand, alveolar ridge preservation may incur higher costs and has a limited effect on increasing soft tissue quality or quantity of the alveolar ridge [34, 35]. Deproteinized bovine BS comprises an inorganic hydroxyapatite-supported structure with osteoconductive properties and protracted resorption kinetics [36–38]. Residual graft material has been detected even a decade post-augmentation, but the extent of osteoclastic activity surrounding bovine BS remains debatable [16, 39, 40]. Given the variety of particulate bovine BS available, it remains to be seen which BS is most suitable for specific clinical scenarios, each with its advantages and limitations.

This narrative review aims to evaluate pre-clinical and clinical data comparing different particulate bovine BS for oral regeneration.

## Methods

A narrative review was conducted by systematically searching the PubMed database up to February 2024, utilizing various combinations of search terms including “particulate”, “bovine”, “bone substitute”, “bone graft”, “pre-clinical”, “clinical”, “in-vitro”, “in-vivo”, “ridge preservation”, “sinus floor elevation”, “peri-implant defects”, “alveolar ridge augmentation”, and “titanium mesh”. Pre-clinical and clinical studies that compared at least two distinct particulate bovine BS were included in the narrative review. Studies that compared particulate bovine BS with other types of particulate BS (e.g., synthetic BS), different particulate xenogeneic BS (e.g., porcine and equine), particulate bovine BS with bovine bone blocks, and identical particulate bovine BS with and without biological substitute modifications (e.g., platelet-rich fibrin, PRF), as well as those that exclusively evaluated a single particulate bovine BS, were excluded. The review focused on general information regarding manufacturing and treatment processes, biological safety, physical and chemical characteristics, and graft resorption of the included particulate bovine BS. Additionally, specific attention was given to pre-clinical data and clinical outcomes related to ridge preservation, sinus floor elevation, peri-implant defects, lateral and vertical alveolar ridge augmentations, and particulate bovine BS for filling titanium meshes.

## Results

### Manufacturing and treatment process of particulate bovine bone substitutes

The primary objective of the treatment process is to eliminate all organic bone components, including proteins, potentially immunologically active substances, and pathogens. Only the mineral bone matrix, particularly hydroxyapatite with an ultra- and nanostructure like human bone, should remain intact. Variations in the manufacturing processes of particulate bovine BS account for differences in material properties even though the origin of the bovine bone remains constant. To produce high-temperature treated bovine BS, the bone raw material undergoes stepwise heating to temperatures exceeding 1,200 °C, followed by multiple cleaning and washing steps using only water. In this regard, heat treatment appears to be the most prevalent preparation method [41]. The elevated temperature offers the advantage of eliminating organic compounds and safely eradicating pathogens such as bacteria, viruses, and prions [42]. In contrast, low-temperature treated bovine BS is prepared through a heat treatment process reaching temperatures up to 300 °C, followed by a chemical cleaning step involving highly concentrated alkaline sodium hydroxide [43]. Due to these distinct processing methods, high-temperature treated bovine BS exhibits highly pure and crystalline hydroxyapatite ceramic characteristics, featuring interconnected pores and a super hydrophilic surface texture. Additionally, its hydroxyapatite crystal size is increased by 500–1,000% compared to human bone. Conversely, low-temperature treated bovine BS displays a texture with hydroxyapatite crystal sizes increased by 200–300% and a more fiber-like structure [43, 44]. Table 1 summarizes different manufacturers’ different low- and high-temperature treated bovine BS.

**Table 1:** Summary of different low- and high-temperature treated bovine bone substitutes (BS) (n.a. = not available)

BS	Manufacturer	Treatment
Bio-Oss®	Geistlich Biomaterials Vertriebsgesellschaft mbH, Baden-Baden, Germany	low-temperature
Cerabone®	botiss biomaterials GmbH, Zossen, Germany	high-temperature
Creos™ xenogain®	Nobel Biocare AG, Kloten, Switzerland	n.a.
MinerOss® X	BioHorizons, Birmingham, AL, USA	n.a.
Osteograf®	DENTSPLY Friadent Ceramed, Lakewood, NJ, USA	high-temperature
NuOss®	ACE Surgical Supply Inc, Brockton, MA, USA	n.a.
Smartbone®	IBI SA Industrie Biomediche Insubri SA, Mezzovico-Vira, Switzerland	n.a.
BonAP®	Legeros, New York University, NY, USA	high-temperature
bonefill®	Bionnovation biomaterials, São Paulo, Brazil	n.a.
GenOx®	Baumer, São Paulo, Brazil	n.a.
Laddec®	BioHorizons, Birmingham, AL, USA	n.a.
InterOss®	SigmaGraft biomaterials, Fullerton, CA, USA	low-temperature
A-Oss®	Osstem Implant, Seoul, Korea	low-temperature
Endobon®	Zimmer Biomet, Warsaw, Indiana, USA	n.a.
Lumina-Bone Porous®	Criteria Biomateriais, São Paulo, Brazil	low-temperature
Biocera®	Oscotec Inc., Gyeongi, Korea	n.a.
Alpha Bio’s Graft®	botiss biomaterials GmbH, Zossen, Germany	high-temperature
InduCera® Dual Coat	Oscotec Inc., Bundang-gu, Seongnam, Gyeonggido, South Korea	n.a.
Osseous®	Sistema de Implantes Nacional, SIN, São Paulo, Brazil	n.a.
Ti-Oss®	Chiyewon, Gyeonggido, South Korea	n.a.
OCS-B®	Nibec, Seoul, South Korea	n.a.

In summary, distinctions must be made between high-temperature and low-temperature treated particulate bovine BS, with both heat and chemical treatment processes utilized depending on the specific product and manufacturer.

### Biological safety of particulate bovine bone substitutes

The fundamental prerequisite for utilizing BS derived from natural sources is thoroughly removing tissue, organic constituents, immunogenic substances, and pathogens to mitigate the risks of disease transmission and adverse reactions. The initial step in ensuring material safety involves carefully selecting donor animals. Bovine bones, particularly femoral heads, sourced from registered slaughterhouses in countries classified as having negligible risk for bovine spongiform encephalopathy (BSE) by the World Organization for Animal Health (OIE), are utilized in the production of both high- and low-temperature treated BS [45, 46]. Another crucial safety measure involves employing approved heat treatment methods independently or with chemical cleaning. It is established that temperatures exceeding 800 °C significantly reduce the risk of transmitting prion diseases, particularly BSE, to an acceptable minimum [47]. Conversely, for low-temperature treated BS, prion inactivation primarily relies on treatment with highly concentrated sodium hypochlorite [45, 48, 49]. In 2011, Kim et al. conducted a systematic review to assess the risk of prion disease transmission associated with bovine BS. Residual protein structures were found in low-temperature treated BS [50], in contrast to high-temperature treated BS [51]. No literature was found regarding the efficacy of BSE prion inactivation during the manufacturing process of bovine BS. However, a patented method in Canada combines sodium hypochlorite and temperatures exceeding 600 °C, claiming to produce a BSE prion-free bovine bone grafting substitute (CA2649970C). In summary, while bovine BS may theoretically pose a risk of transmitting BSE prions, the current available information does not allow for quantification of this risk [52]. From a practical-clinical standpoint, there have been no reported instances of disease transmission through bovine BS, suggesting that their application is unlikely to result in transmission complications [43].

### Physical and chemical characteristics of particulate bovine bone substitutes

As per the manufacturer specifications, the particle size of high-temperature treated bovine BS ranges from 500 to 1,000 μm, whereas that of low-temperature treated BS ranges from 250 to 1,000 μm. Literature on human bone indicates an average particle size of approximately 280 μm [53]. High-temperature treated BS exhibits a porosity of 72%, whereas low-temperature treated BS ranges from 75 to 80% [54]. Both bovine BS formulations consist of crystalline components and possess calcium/phosphate ratios ranging from 1.75 to 1.33. Both variants demonstrate the release of calcium ions, albeit at lower levels than human bone (high-temperature treated BS: 4 mg/g; low-temperature treated BS: 12 mg/g; human bone: 20 mg/g) [53]. In addition to porosity, pore sizes are noteworthy, with a systematic review reporting pore sizes of bovine BS ranging from 1.3 to 1,000 μm [41, 55]. In brief, there is a notable heterogeneity in the characteristics of different bovine BS, particularly in porosity, crystallinity, and calcium/phosphate ratio [41]. Biomechanical properties of bovine BS are also affected by rehydration. In the case of bovine BS blocks, high-temperature treated BS demonstrated limited height reduction following rehydration compared to dry conditions, with no alteration in rigidity [56]. Regarding hydrophilicity, high-temperature treated BS exhibited increased hydrophilicity compared to low-temperature treated BS [56].

### Resorption of particulate bovine bone substitutes

Ideally, bovine BS undergoes gradual remodeling into newly formed bone through a process known as “creeping substitution” [57]. For low-temperature treated BS, a minimal or complete absence of resorption is postulated [36, 58–60] with similar assumptions for high-temperature treated BS, where a prolonged resorption rate may be anticipated, if any [61]. Nonetheless, comparable stability in maxillary sinus volumes following sinus floor elevation with either low- or high-temperature treated BS has been observed [57, 62], suggesting similar resorption characteristics between the two BS. In conclusion, both low- and high-temperature treated bovine BS demonstrate minimal or negligible degradation within the body, thus qualifying them as potential permanent substitutes for bone regeneration and integration. A study assessing circumferential defect filling around implants in minipigs with either high- or low-temperature treated BS revealed equivalent outcomes regarding graft resorption and new bone formation at 8 and 12 weeks post-implantation [63].

### Pre-clinical studies

#### Pre-clinical in vitro data

Concerning pre-clinical in vitro data, a study examined the effects of various particulate bovine, synthetic, and allogeneic BS (cerabone® (botiss biomaterials GmbH, Zossen, Germany), Bio-Oss® (Geistlich biomaterials Vertriebsgesellschaft mbH, Baden-Baden, Germany; bovine each), maxresorb® (botiss biomaterials GmbH; synthetic), maxgraft® (botiss biomaterials GmbH; allogeneic)) and their combination with platelet-rich fibrin (PRF) on various cell characteristics (cell viability, proliferation rate, and migration ability) of osteoblasts after 24 h in vitro. No differences were observed between the two tested particulate bovine BS under native conditions [26]. Another study addressed PRF-biofunctionalization, investigating the potential benefits of injectable PRF (iPRF) on four different particulate bovine BS (cerabone®; Bio-Oss®; creos™ xenogain® (Nobel Biocare AG, Kloten, Switzerland); MinerOss® X (BioHorizons, Birmingham, AL, USA)) and its impact on osteoblasts in vitro. Cell viability, metabolic activity, and gene expressions (alkaline phosphatase, ALP; osteocalcin, OCN; and bone morphogenetic protein-2, BMP-2) were analyzed at measurement timepoints after 3, 7, and 10 days. For native, non-iPRF modified BS, cerabone® exhibited the highest cell viability at all time points, while MinerOss® X showed the most increased metabolic activity after 3 days and cerabone® after 7 and 10 days. Overall, the authors observed a clear trend of favorable outcomes regarding cell viability and metabolic activity for high-temperature treated particulate bovine BS compared to low-temperature treated ones [29]. Another in vitro study investigated the effects of low-temperature (Bio-Oss®) and high-temperature treated BS (Osteograf®, DENTSPLY Friadent Ceramed, Lakewood, NJ, USA) and high-temperature bovine hydroxyapatite modified with p-15 on osteoblast cell viability, proliferation rate, and cell morphology. Osteograf® demonstrated higher proliferation rates and improved cell differentiation than Bio-Oss®, as confirmed by scanning electron microscopy [64]. In terms of optimal treatment temperatures, a study evaluated three different temperature durations and temperatures (3 h at 300 °C, 3 h at 300 °C followed by 6 h at 530 °C, and 3 h at 300 °C followed by 2 h at 1000 °C) and Bio-Oss®, on bone marrow stromal cells (migration, proliferation, differentiation) in vitro. In addition, calvaria defects in rabbits were employed to analyze bone regeneration and graft degeneration. The study found that 3 h at 300 °C followed by 6 h at 530 °C induced the highest cell attraction and improved cell differentiation, while both 3 h at 300 °C and 3 h at 300 °C followed by 6 h at 530 °C resulted in higher bone fraction after 6 and 12 weeks in vivo [65]. Ortiz-Puigpelat investigated the blood absorption of different bovine BS and demonstrated a significantly increased blood absorption of NuOss® (ACE Surgical Supply Inc, Brockton, MA, USA) and Bio-Oss® compared to Smartbone® (IBI SA Industrie Biomediche Insubri SA, Mezzovico-Vira, Switzerland) [66].

In summary, pre-clinical in vitro data may provide slight evidence of minor advantages of high-temperature treated BS, such as improved biocompatibility in cell cultures.

### Pre-clinical in vivo data

In terms of pre-clinical in vivo data, synthetic calcium phosphate and two particulate bovine BS (low-temperature treated Bio-Oss®, high-temperature treated BonAP® (Legeros, New York University, NY, USA)) were implanted into the femoral epiphysis of rats and evaluated after three weeks. The bone ingrowth for the synthetic calcium phosphate was higher due to its increased resorption rate [67]. A recent review by Peric Kacarevic et al. synthesized findings from eight studies focusing on low-temperature treated BS (Bio-Oss®, seven studies in the rabbit calvaria model and one in sheep), three studies examining high-temperature treated BS (cerabone®, two studies in rabbit and rat calvaria models and one periapical study in cats), and one comparative study between high and low-temperature treated BS (cerabone® and Bio-Oss®) in the rabbit calvaria model, yielding overall comparable results. Depending on the time, new bone formation rates ranged from 0 to 57%. After 14–30 days, Bio-Oss® demonstrated an average new bone formation rate of 19%, whereas cerabone® exhibited 20%. After 42–84 days, Bio-Oss® showed an average new bone formation rate of 24%, while cerabone® displayed 47%. Finally, after 112–168 days, Bio-Oss® demonstrated an average new bone formation rate of 43%, whereas cerabone® exhibited 26% [43]. In a study by Manfro et al., three bovine BS (Bio-Oss®, bonefill® (Bionnovation biomaterials, São Paulo, Brazil), and GenOx® (Baumer, São Paulo, Brazil)) were analyzed, with blood clot augmentation as a control. Histomorphometric analysis revealed that Bio-Oss® and Bonefill® exhibited greater new bone formation compared to GenOx® and blood clots after 8 and 12 weeks [68]. Conversely, in a recent study utilizing the rat calvaria model to compare Bio-Oss® and Bonefill® with blood clots, no significant differences were observed between the two bone substitutes and the control group without bone substitutes [69]. Another investigation in the rabbit calvaria model compared Laddec® (BioHorizons, Birmingham, AL, USA) with Bio-Oss® for the augmentation of critical 8 mm defects. Both materials demonstrated comparable and complete defect regeneration with slow resorption rates at 4 and 8 weeks, with Bio-Oss® augmented defects exhibiting less soft tissue migration [70]. A study investigated the characteristics of different particulate bovine BS using a minipig model with bilaterally induced mandibular bony defects (total *n* = 72). The study group design comprised a control group without BS, low-temperature treated BS (Bio-Oss®), and high-temperature treated BS (cerabone®), both combined with a porcine pericardium membrane. Harvesting and micro-CT analysis was conducted at 4-, 8-, and 12-weeks post-implantation. No volumetric differences were observed among the groups after 4 weeks. However, at 8 and 12 weeks, the control group and cerabone® exhibited a significantly higher percentage of new bone formation compared to Bio-Oss® [71]. In another study, the same BS (Bio-Oss® and cerabone®) were compared for sinus floor augmentation in rabbits. Histometric and micro-CT analyses indicated that both biomaterials facilitated bone growth within the elevated space, with no significant differences observed between the two BS. Consequently, the authors concluded that both biomaterials are equally suitable for sinus floor augmentation [72]. Subsequently, critical-sized alveolar ridge defects in canines (*n* = 27 animals, total *n* = 54 defects) were filled with either InterOss® (SigmaGraft biomaterials, Fullerton, CA, USA) or Bio-Oss® (both low-temperature treated), while empty, non-filled defects served as controls. Data analysis was conducted at 4-, 8-, and 12-weeks post-implantation. No significant differences were observed between the experimental groups regarding graft resorption, bony integration, percentage of mineralized bone volume, and density based on radiographic analysis. Histologic examination similarly revealed no discernible differences between the two groups, although there was a non-significant trend towards increased bone formation for InterOss® compared to Bio-Oss® after 8 and 12 weeks [73]. In another study, bone level tapered implants (BLT) were inserted into the mandibles of 10 minipigs, where circumferential defects measuring 4 mm in depth and 2 mm in width were created around the implants. These defects were filled with particulate bovine BS (cerabone®, Bio-Oss®). Harvesting and data analysis were conducted at 8- and 12-weeks post-implantation. No significant differences were detected regarding new bone formation, bone substitute resorption, or bone-implant contacts, with similar outcomes observed across both groups [63]. In a separate investigation, alveolar defects were induced in 6 beagles and filled with either InterOss® or A-Oss® (Osstem Implant, Seoul, Korea; both low-temperature treated) using GBR four weeks post-defect creation. Both BS resulted in new bone formation, with no significant disparities observed in histological, histomorphometric, and volumetric analyses [74].

In summary, pre-clinical in vivo data failed to elucidate substantial variances between the various particulate bovine BS. However, a subtle tendency towards variances in new bone formation between low- and high-temperature treated bovine BS was observed across distinct healing intervals, with potential slight benefits favoring high-temperature treated bovine BS after 2–3 months.

### Clinical studies

#### Particulate bovine bone substitutes for ridge preservation

In the context of ridge preservation, a prospective, single-center randomized study compared two bovine BS (Bio-Oss®; Endobon®, Zimmer Biomet, Warsaw, Indiana, USA) when applied with a resorbable membrane. This study involved 60 patients and 40 extraction sites, with 20 sites allocated to each bovine BS. Histological analysis was performed after 4 months, coinciding with implant placement. The results indicated no differences in new bone formation between the two tested particulate bovine BS. There were no significant differences in implant survival rate, which was 100% for Bio-Oss® and 95% for Endobon® after 24 months follow-up [75]. Furthermore, a multicenter prospective randomized controlled trial (RCT) compared two different particulate bovine BS, one high-temperature treated (Endobon®) and the other low-temperature treated BS (Bio-Oss®), for ridge preservation. The study involved 38 patients and a total of 62 augmented extraction sites. Defects were covered with resorbable collagen membranes, and re-evaluation occurred after 6 months in conjunction with implant placement. Histologic evaluations were performed for data analysis, revealing similar outcomes between the two bovine BS, with comparable levels of new bone formation (28.5 ± 20% and 31.4 ± 18%) [76]. In a RCT by Shakibaie et al., Bio-Oss® and cerabone® were compared at 20 adjacent extraction sites in 10 patients. Successful implant therapy was achieved at all extraction sites, regardless of the BS. However, the Bio-Oss® group exhibited superior outcomes in terms of crestal gingival healing process (CGHP), mean transversal crestal ridge resorption (MTRR), and mean implant primary stability (MIPS) [77].

In summary, the existing data do not suggest significant differences regarding the efficacy of different particulate bovine BS for ridge preservation. A RCT showed marginal benefits of low-temperature treated bovine BS over high-temperature treated counterparts in ridge preservation, specifically in crestal gingival healing, transversal crestal ridge resorption, and primary implant stability. However, the available data are inadequate to support an evidence-based conclusion.

### Particulate bovine bone substitutes for sinus floor elevation

**Fig. 1:**
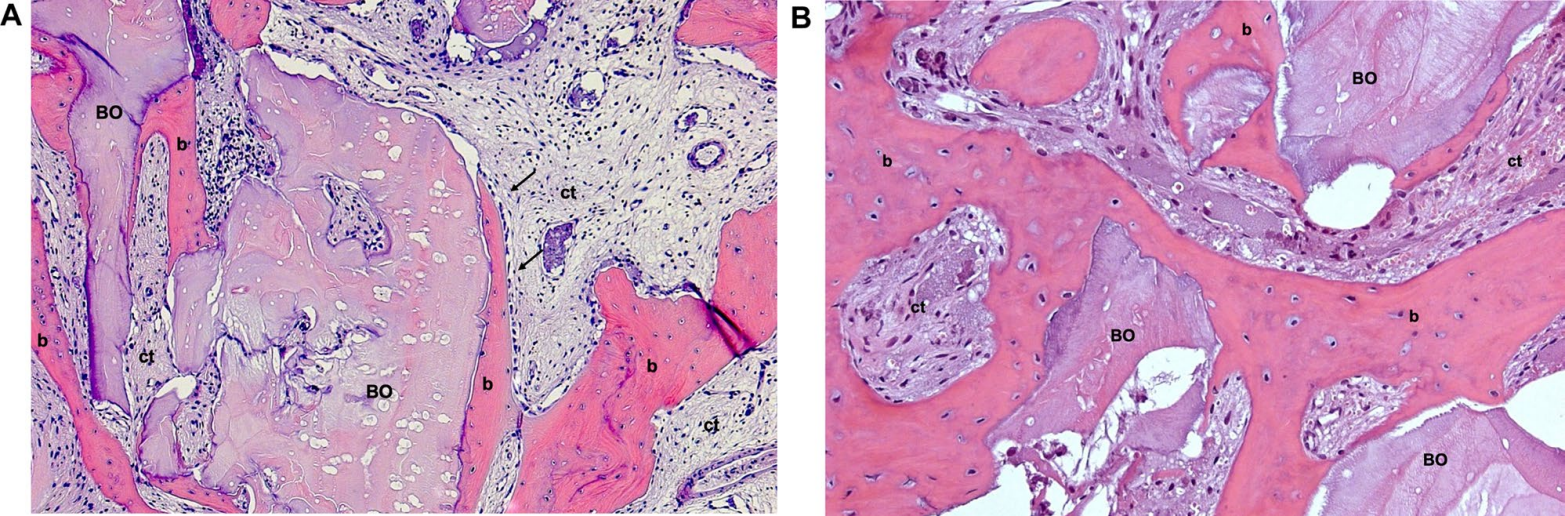
Histological sections (H&E-stained) 5–8 months after sinus floor elevation with a low-temperature treated bovine BS (Bio-Oss®). **(A**) Early osteogenesis around bone substitute (BS) granules forming woven bone (b), ct = connective tissue, black arrows = osteoblasts; original magnification (o.m.) 20-fold. **(B**) Advanced osteogenesis around BS residual granules, b = newly formed woven bone, ct = connective tissue; o.m. 20-fold

**Fig. 2:**
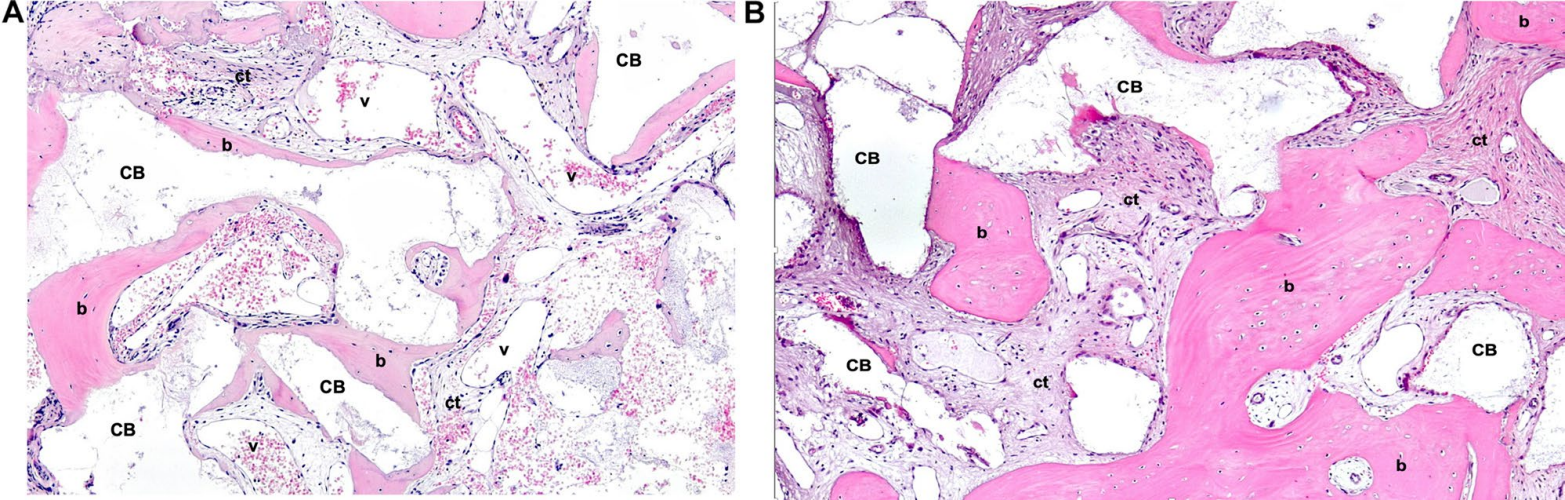
Histological sections (H&E-stained) 5–8 months after sinus floor elevation with a high-temperature treated bovine BS (cerabone®). **(A**) Early osteogenesis around bone substitute (BS) granules forming woven bone (b), granules mostly lost due to decalcification, ct = highly vascularized connective tissue, v = vessels; original magnification (o.m.) 10-fold. **(B**) Advanced osteogenesis around BS granules forming woven bone (b) partly remodeling into lamellar bone, granules mostly lost due to decalcification, ct = connective tissue; o.m. 10-fold

**Fig. 3:**
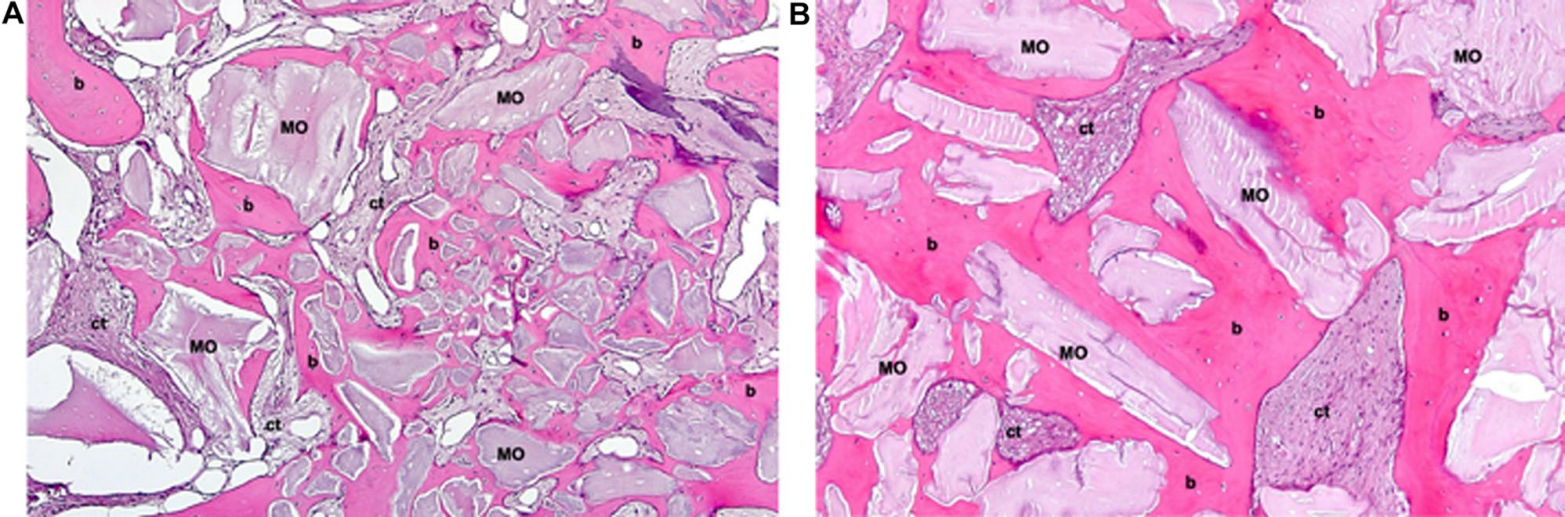
Histological sections (H&E-stained) 5–8 months after sinus floor elevation with a bovine BS (MinerOss®). **(A**) Early osteogenesis around cluster of small bone substitute (BS) granules forming woven bone (b), ct = connective tissue; original magnification (o.m.) 10-fold. **(B**) Advanced osteogenesis around larger BS granules forming woven bone (b), ct = connective tissue; o.m. 10-fold

Peric Kacarevic et al. synthesized bone formation rates observed in elevated sinuses from clinical studies, which, in line with preclinical analyses, exhibited similarity between the included bovine BS (Bio-Oss® and cerabone®) [43]. However, only two clinical trials investigated two different bovine BS (Bio-Oss® and cerabone®) for sinus floor elevation over 8 months and 4 years. One of these studies reported a significantly higher radiographically determined volume loss after four years with Bio-Oss® compared to cerabone® (33.4 ± 3.1% and 23.4 ± 3.6%, respectively). Another study demonstrated an increased rate of new bone formation for cerabone® (29.13%) compared to Bio-Oss® (24.63%) [43, 57, 62]. Additionally, another study analyzed the osteoconductivity index (OI) of Bio-Oss® and cerabone® through histological examination and micro-CT scans, quantifying the contact length between the particulate bovine BS and the newly formed bone as a percentage. Twenty patients underwent sinus floor elevation, with the resultant voids filled using either cerabone® or Bio-Oss®. Biopsies were obtained after 5 months in conjunction with implant placement. Micro-CT scans revealed comparable OI between the two groups (cerabone®: 92.72 ± 8.77%, Bio-Oss®: 88.84 ± 8.81%). Regarding tissue distribution assessed via micro-CT scans, no significant differences were observed. Cerabone® exhibited increased bone tissue (50.12 ± 10.97%), decreased substitute remnants (16.12 ± 10.11%), and less connective tissue (33.77 ± 10.81%) compared to Bio-Oss® (44.02 ± 10.48%, 18.06 ± 12.53%, 37.92 ± 8.28%, respectively). Histologically, cerabone® displayed decreased bone tissue (20.4 ± 8.22%), increased substitute remnants (29.82 ± 11.75%), and less connective tissue (49.78 ± 10.37%) compared to Bio-Oss® (23.95 ± 8.89%, 23.47 ± 8.26%, 52.57 ± 3.89%, respectively), albeit without statistical significance [46]. A comparative study assessed sinus floor elevation using Bio-Oss® and Lumina-Bone Porous® (Criteria Biomateriais, São Paulo, Brazil) in a split-mouth model involving 13 patients. Lumina-Bone Porous® is a xenogeneic BS processed without high temperatures and undergoes chemical sterilization instead. Clinical, radiological, and histological evaluations were conducted. Both bovine BS induced significant bone gain in elevated sinuses after 6 months compared to baseline. Histologically, no significant differences in newly formed bone were observed between the two particulate bovine BS. However, Bio-Oss® exhibited significantly more residual BS material [78]. In another study, the efficacy of Biocera® (Oscotec Inc., Gyeongi, Korea) and Bio-Oss® for sinus floor elevation was compared in five patients with elevation procedures performed on both maxillary sinuses. Histological assessment was carried out after 6 to 8 months. Results revealed minimal, non-significant differences in new bone formation between Bio-Oss® (28.46 ± 5.28%) and Biocera® (29.94 ± 8.72%) [79]. A prospective study examined histological aspects of new bone formation following sinus floor elevation using Bio-Oss® and cerabone®. The study enrolled 22 patients with approximately 3 mm residual bone height who underwent two-stage implant placements. Histological analysis after 6 months revealed no differences between new bone formation and residual BS material [40]. In a RCT, a comparison was made between a high-temperature treated particulate bovine BS (Alpha Bio’s Graft®, a private label of cerabone®, botiss biomaterials GmbH, Zossen, Germany) and a low-temperature treated one (Bio-Oss®) for sinus floor elevation. After 6 months, similar augmentation heights (> 95%) and percentages of new bone (> 30%) were observed, with a slightly reduced amount of residual bone in the high-temperature treated group (40.68 ± 16.32%) compared to the low-temperature treated group (43.43 ± 19.07%). However, the differences were not significant [80]. Canellas et al. conducted a systematic review and meta-analysis to evaluate various xenogeneic BS for sinus floor elevation. Eleven studies were included in the analysis, focusing on histological assessments of newly formed bone and graft residues. Among the comparisons involving particulate bovine BS, Bio-Oss® exhibited significantly higher graft residues compared to Lumina-Bone Porous® (Criteria Biomateriais, São Paulo, Brazil) (19.9% vs. 14.6%, *p* = 0.015) The survival rate of dental implants for augmented area with Lumina Bone Porous® was 88.88%, while for Bio-Oss® group was 100% at 3-year follow-up after final prosthetic restoration [78]. No significant differences were found between Bio-Oss® and InduCera® Dual Coat (Oscotec Inc., Bundang-gu, Seongnam, Gyeonggi-do, South Korea) [81]. Similarly, no significant differences were observed when comparing Osseous® (Sistema de Implantes Nacional, São Paulo, Brazil) and Bio-Oss®, either in newly formed bone or graft residues [82, 83]. In a retrospective study, 36 patients with a ridge height of less than 4 mm in the posterior maxilla underwent sinus floor elevation using three different BS: Bio-Oss®, cerabone®, and Ti-Oss® (Chiyewon, Gyeonggido, South Korea). Biopsies were obtained for histological and histomorphometric analysis at six months postoperatively, and volumetric measurements were conducted one week and six months post-surgery. The Ti-Oss® group exhibited inferior outcomes compared to the Bio-Oss® and cerabone® groups, displaying significantly higher levels of bone resorption and less new bone formation. Additionally, all groups experienced a significant reduction in graft volume between one week and six months post-surgery [84]. Hieu et al. radiographically compared the height of the Bio-Oss® and OCS-B® (Nibec, Seoul, Korea) graft materials after sinus floor elevation and found that a smaller amount of Bio-Oss® maintained greater absolute volume over time compared to OCS-B®. There was no significant change in the Bio-Oss® group, while the OCS-B® group showed a significant decrease in bone height up to a follow-up period of 25 months, with no significant differences found when comparing both BS [85]. Figures 1, 2 and 3 present histological sections from biopsies after sinus floor elevations with three different bovine BS after healing times ranging from 5 to 8 months.

In summary, the data gleaned from individual clinical studies indicate subtle clinical distinctions among various particulate bovine BS for sinus floor elevation in certain aspects. In brief, low-temperature treated BS tented to an increased long-time bone volume loss. High-temperature-treated BS seemed to improve new bone formation and decrease the amount of connective tissue. The potential differences between bovine-derived BS are only evident or by trend in individual studies and do not permit evidence-based conclusions.

### Particulate bovine bone substitutes for peri-implant defects

In a comparative study, EndoBon® and Bio-Oss® were evaluated for filling peri-implant defects in 24 patients diagnosed with peri-implantitis. Various parameters, including bone level, intrabony defect depth, probing depth, bleeding on probing, and suppuration on probing, were assessed at 6- and 12-months post-treatment. Both bovine BS significantly improved all measured parameters at both time points. However, no significant differences were observed between the two groups for any parameter at any time point [86].

The available data does not suggest relevant differences between different particulate bovine BS for treating peri-implant defects. An evidence-based assessment is not possible due to the available data.

### Particulate bovine bone substitutes for lateral and vertical ridge augmentation

Lim et al. conducted a comparative study to evaluate two low-temperature treated bovine BS, Bio-Oss® and A-Oss® (Osstem Implant, Prague, Czechia) for lateral augmentation during simultaneous implant insertion. The study included eight patients in each group. Implants were placed concurrently with the augmentation using a double-layer technique involving internal allograft and external xenogeneic BS, covered by a preformed ultrafine titanium mesh and a resorbable collagen membrane. All implants were successfully prosthetically treated. After a twelve-month follow-up period, both groups exhibited graft volume shrinkage, with a reduction of 46% in the Bio-Oss® group and 40.8% in the A-Oss® group. However, no significant differences were observed in graft volume changes over time or in bone density between the two groups [87].

The available data does not suggest relevant differences between different particulate bovine BS for lateral and vertical ridge augmentation. Due to the current state of available data, a definitive evidence-based assessment is unfeasible.

### Particulate bovine bone substitutes for titanium mesh filling

No studies were found comparing different particulate bovine BS for titanium mesh filling.

## Conclusions

This narrative review identified a shortage of data directly comparing various particulate bovine BS in oral regeneration. Regarding preclinical data, high-temperature treated particulate bovine BS may offer slight advantages, such as enhanced in vitro biocompatibility compared to low-temperature treated counterparts. Regarding sinus floor elevation, there is a trend towards small benefits with high-temperature treated particulate bovine BS regarding volume loss, new bone formation and the amount of connective tissue. It warrants acknowledgment that pivotal clinical metrics including implant survival, implant success, and ridge volume preservation frequently remain unexplored within existing literature. While many publications focus on the assessment of newly formed bone, an indispensable aspect, such analysis alone fails to comprehensively evaluate the clinical efficacy of BS. No significant differences were noted for other clinical indications, and no specific recommendations favoring specific particulate bovine BS could be made. Further clinical investigations are urgently required to address this data gap and compare different particulate bovine BS across various clinical indications.

## Data Availability

No datasets were generated or analysed during the current study.
